# Clinical Features and Outcome of 79 Dogs With Digital Squamous Cell Carcinoma Undergoing Treatment: A SIONCOV Observational Study

**DOI:** 10.3389/fvets.2021.645982

**Published:** 2021-04-30

**Authors:** Laura Marconato, Daniela Murgia, Riccardo Finotello, Valeria Meier, Emanuela Maria Morello, Luciano Pisoni, Armando Foglia, Dina Guerra, Carmit Chalfon, Marina Aralla, Paola Mesto, Maurizio Annoni, Francesco Albanese, Giuliano Bettini, Silvia Sabattini

**Affiliations:** ^1^Department of Medical Veterinary Sciences, University of Bologna, Bologna, Italy; ^2^Dick White Referrals, Six Mile Bottom, United Kingdom; ^3^Department of Small Animal Clinical Science, Institute of Infection, Veterinary, and Ecological Science, University of Liverpool, Liverpool, United Kingdom; ^4^Division of Radiation Oncology, Small Animal Department, Vetsuisse Faculty, University of Zurich, Zurich, Switzerland; ^5^Department of Veterinary Sciences, University of Turin, Turin, Italy; ^6^Pronto Soccorso Veterinario Laudense, Lodi, Italy; ^7^Centro Medico Veterinario BMVET, Bari, Italy; ^8^AniCura Clinica Veterinaria Tibaldi, Milan, Italy; ^9^Laboratorio di Analisi Veterinarie LaVallonea, Rho, Italy

**Keywords:** squamous carcinoma, digit, dog, metastasis, Schnauzer

## Abstract

In dogs, digit squamous cell carcinoma (SCC) is uncommon. Clinical signs are frequently underestimated, leading to a diagnostic delay. The purpose of this retrospective study was to report our experience regarding the clinical presentation, diagnostic work-up, treatment and outcome of 79 client-owned dogs with SCC of the digit. The greatest majority (84.8%) of dogs was dark-coated. Schnauzers represented approximately one third of the study population, and had a poorer outcome compared with other breeds. The majority of SCCs occurred in the front limbs (61%), and bone lysis was frequently observed (92.4%). Approximately 9% of dogs had involvement of multiple digits, and this was associated with a shorter time to progression (TTP; *P* = 0.047). Similarly, a duration of clinical signs >90 days was associated with a shorter TTP (*P* = 0.02). Regional lymph node metastases were documented in 17.7% of dogs at admission and were significantly associated with tumor-related death (*P* < 0.001). At presentation, none of the dogs had evidence of distant metastasis. Digit amputation achieved adequate local tumor control in the majority of cases. Adjuvant chemotherapy and radiation therapy were carried out in 21.5% of cases, with uncertain benefit. Due to the relatively non-aggressive clinical behavior of digit SCC, chemotherapy should only be offered in the case of metastatic disease. Approximately one fourth of dogs developed *de novo* SCCs during the follow-up. Careful examination of the digits should be encouraged in breeds considered at high risk and in dogs with a previous history of digital SCC.

## Introduction

Cancer of the digits is considered to be uncommon in dogs, with squamous cell carcinoma (SCC) representing the main histotype (1, 2).

While exposure to ultraviolet radiation of poorly pigmented skin has been associated with the cutaneous form of canine and feline SCC, no predisposing factors have been described for digital SCCs (3). In humans, Papillomaviruses (PVs) are an established cause of cancer (4), and there is accumulating evidence that they may be responsible for the development of oral and cutaneous SCCs in cats (5, 6). In this regard, several studies have investigated the relationship between PV and canine SCC with contradictory results (7, 8). To date, the causative role of PV cannot be totally excluded.

Clinical signs of digital SCC mainly include digital swelling and lameness, but SCC may mimic a wide variety of other diseases, such as bacterial, fungal or protozoal pyogranulomatous infections, epithelial inclusion cysts, infundibular keratinizing acanthoma, and other benign tumors (9). This frequently causes misdiagnosis and late presentation, possibly contributing to an underestimation of its incidence. Referral to a specialist is often made only after failure of medical management or repeated surgical attempts by first opinion veterinarians.

Digital SCC usually affects large breed dogs with dark hair coat (10), with Standard Poodles, black Labrador Retrievers, Giant Schnauzers, Gordon Setters, Rottweilers, Beaucerons, and Briards being pre-disposed to multiple lesions (10–12).

Bone lysis occurs in 80% of dogs (1), and digit amputation is therefore the treatment of choice (1, 13). Nodal or distant metastasis at admission is reported in <15% of dogs (1, 14); however, no recommendation regarding adjuvant medical treatment is currently available.

According to the published studies, dogs undergoing digit amputation will have a 1- and 2-year survival rates of 50–83% and 18–62%, respectively (1, 2, 14). The wide range of survival rates translates into an objective difficulty in communicating with the owner. In one study, dogs with SCC arising from the subungual epithelium experienced a better outcome (14), with 1- and 2-year survival rates of 95 and 74%, respectively.

Because of the rarity of this cancer, there are only a few clinical studies that focused specifically on digital SCC, and the latest dates back to 2007 (2). Since then, no changes regarding staging work-up and treatment recommendations have been implemented.

The aim of this retrospective observational study was to report our experience regarding the clinical presentation, diagnostic work-up, treatment and outcome of 79 dogs with digital SCC.

## Materials and Methods

Members of the Italian Society of Veterinary Oncology (SIONCOV) were asked to retrospectively search their records to identify dogs with newly diagnosed digital SCC without any previous surgery or cytotoxic treatment history for the disease. To be enrolled, dogs had to have a complete staging work-up, including regional lymph node assessment (either by cytology or histology), thoracic radiographs, digit radiographs, and abdominal ultrasound or total body computed tomography (TBCT), have undergone surgery, and have a complete follow-up assessed by clinical examinations or phone calls to the referring veterinarians or owners. Adjuvant cytotoxic treatment was allowed and documented. Medical records had to be sufficient to assess treatment modalities and to evaluate treatment response (15).

For each dog, the following data were recorded: signalment (breed, hair coat color, sex, age, weight), clinical signs and duration of signs, whether the lesion involved one or multiple digits, site of involvement, ulceration, bone lysis, type of staging work-up performed, type of treatment, clinical response to treatment, time to progression (TTP), time to new digital lesions (TDG), survival time (ST), and cause of death.

## Statistical Analysis

Descriptive statistics were used in the analysis of dogs and tumor characteristics. When appropriate, data were tested for normality by use of the D'Agostino and Pearson omnibus normality test. Values were expressed as mean ± SD in case of normal distribution, or as median with a range in case of non-normal distribution.

TTP was calculated from the date of surgery to the to the date of first-documented local recurrence or metastasis, whichever occurred first. TDG was calculated from the date of surgery to the date of development of a new digital SCC not contiguous to the previous location but occurring in another digit. Dogs with no events at the date of the last visit or death were censored.

ST was calculated from the date of surgery to the date of death or to the date of the last visit if death did not occur. Only dogs deceased for SCC-related causes were considered as events.

Survival plots were generated according to the Kaplan-Meier product-limit method. Survival estimates were presented as medians with the corresponding 95% confidence intervals (95% CI). The influence of potential prognostic variables on tumor progression, *de novo* SCC development or SCC-related death was investigated with Cox's regression analysis.

The following variables were investigated for prognostic significance: breed (Schnauzers vs. others), sex, age (median used as cut-off), weight (median used as cut-off), clinical signs duration (median used as cut-off), number of lesions at presentation (single or multiple), limb (forelimb or hindlimb), subungual location (yes or no), ulceration (yes or no), largest tumor diameter (median used as cut-off), bone lysis (yes or no), metastases at diagnosis (yes or no), lymphadenectomy (yes or no) and adjuvant medical treatment (yes or no).

Statistical analysis was performed with SPSS Statistics v.19 (IBM, Somers, New York). Significance was set at *P* ≤ 0.05.

## Results

### Patient and Tumor Characteristics

Medical records of dogs with digital SCC between January 2008 and December 2018 at 8 institutions were evaluated. A total of 79 dogs fulfilled the inclusion criteria and were included in the study (*n* = 27 at University of Bologna; *n* = 17 at University of Turin; *n* = 9 at Dick White Referrals; *n* = 9 at Clinica Veterinaria Tibaldi; *n* = 7 at University of Zurich; *n* = 5 at University of Liverpool; *n* = 3 at Centro Veterinario BMVET; and *n* = 2 at Pronto Soccorso Veterinario Laudense).

Demographic information is reported in Table 1.

**Table 1:** Summary of relevant patient and tumor characteristics observed in 79 dogs with digital squamous cell carcinoma.

Sex	
Male intact	35 (44.3%)
Male neutered	15 (19%)
Female intact	11 (13.9%)
Female neutered	18 (22.8%)
Breeds	
Mixed-breed	9 (11.4%)
Schnauzer	25 (31.6%)
Rottweiler	8 (10.1%)
Labrador retriever	7 (8.9%)
Cocker spaniel	4 (5.1%)
Poodle	3 (3.8%)
Other	23 (29.1%)
Hair color^*^	
Dark-coated^**^	67 (89.3%)
Other	8 (10.7%)
Median age (range)	10 (2–18) years
Median weight (range)	33 (6.5–52) kg
Median duration of symptoms (range)	90 (2–700) days
Presentation	
Single	72 (91.1%)
Multiple	7 (8.9%)
Median tumor diameter (range)	2.3 (1–6) cm
Ulceration^*^	
Yes	47 (61.8%)
No	29 (38.2%)
Bone lysis	
Yes	73 (92.4%)
No	6 (7.6%)
Subungual^*^	
Yes	57 (75%)
No	19 (25%)
Tumor differentiation	
Well-differentiated	74 (93.7%)
Moderately differentiated	1 (1.3%)
Poorly differentiated	4 (5%)
Metastasis at presentation	
Yes	12 (15.2%)
No	67 (84.8%)
Development of metastatic disease during the follow-up	
Yes	14 (17.7%)
No	65 (82.3%)
1-year survival rate	81%
2-year survival rate	60%

* *Data non available for all dogs.*

** *Black, brown, black and brown, black and tan*.

Reasons for referral were lameness (*n* = 46, 58.2%), digital swelling (*n* = 22; 7.8%), breaking or splitting of the toenail (*n* = 8; 12.5%), pain (*n* = 5; 6.3%), licking at the digit (*n* = 6; 7.5%), and bleeding from the digit (*n* = 4; 5%). Furthermore, 2 (2.5%) dogs had cutaneous metastatic nodules along the lymphatic vessels.

Median symptom duration was 90 days (range, 2–700 days).

At admission, 72 (91.1%) dogs had a single digit involved, whereas 7 (8.9%) had involvement of multiple digits.

Among dogs with a single SCC, tumors occurred on the forelimb digits in 44 (61.1%) dogs and on the hindlimb digits in 28 (39.4%) dogs. Among dogs with multiple SCC, tumors occurred in one forelimb and in one hindlimb in 5 (71.4%) dogs, and in two hindlimbs in 2 (28.6%) dogs.

When considering all SCCs (*n* = 86), the tumors were distributed as follows: 21 (24.4%) on the second digit, 21 (24.4 %) on the third, 17 (19.8%) on the fifth, 14 (16.3%) on the fourth, and 13 (15.1%) on the first. Median tumor diameter was 2.3 cm (range, 1–6 cm).

Forty-seven (61.8%) tumors were ulcerated, while 29 (38.2%) were not. This information was not available for 3 (3.8%) dogs.

All dogs underwent cytology of the regional lymph node, and 4 (5.1%) had evidence of metastatic disease. Two (2.5%) dogs had metastatic nodules along the lymphatic vessels confirmed by cytology.

In regard to the diagnostic imaging work-up, 69 (87.3%) dogs underwent thoracic radiographs and abdominal ultrasound, and 10 dogs (12.7%) had a TBCT performed. None had evidence of distant metastasis.

All dogs underwent either radiographs or CT of the affected digit, and bone lysis was observed in 73 (92.4%) of them.

### Treatment and Outcome

Surgery was the primary treatment for all dogs. Seventy-two dogs with a single lesion involvement underwent digit amputation (*n* = 71) or leg amputation due to the extensive soft tissue involvement (*n* = 1). Seven dogs with multiple lesions underwent amputation of the affected digits.

Surgical and histological margins were considered complete in all cases.

Based on the histopathology report, 74 tumors (93.7%) were well-differentiated; 1 (1.3%) was moderately differentiated and 4 (5%) were poorly-differentiated. In 57 (75%) dogs, the ungual bed was defined to be the origin of the tumor.

Concurrent lymphadenectomy was performed in 39 (49.4%) dogs. Histopathological evaluation revealed lymph node metastasis in 10 (25.6%) dogs, including those 4 with cytological evidence of metastatic disease. Among these 10 dogs, 2 (20%) had non-subungual SCC.

The overall metastatic rate at the time of presentation was 15.2% (12/79: 10 nodal and 2 cutaneous).

Seventeen (21.5%) dogs received some form of adjuvant treatment: 14 (82.4%) dogs were treated with chemotherapy, 2 (11.8%) with radiation therapy and 1 (5.9%) with both.

Chemotherapy protocols varied widely. Six dogs were treated with weekly intravenous 5-Fluorouracil at the dose of 150 mg/m^2^ with a median number of 10 doses (range, 3–27). Five dogs were treated with an intravenous platinum compound, consisting of carboplatin (300 mg/m^2^ every 21 days for 4 cycles; *n* = 3) or cisplatin (60 mg/m^2^ every 21 days for 3 cycles; *n* = 2). Three (21.4%) dogs were treated with oral metronomic chemotherapy consisting of thalidomide (4 mg/kg daily), cyclophosphamide (10 mg/m^2^ daily), and piroxicam (0.3 mg/kg daily). Median treatment duration in these dogs was 6 months (range, 4–7 months).

Dogs undergoing radiation therapy received 3 × 8 Gy fractions (*n* = 2) or 12 × 4 Gy fractions (*n* = 1). One of them also received carboplatin at 250 mg/m^2^ for 4 cycles.

The median follow-up time was 614 days (range, 128–2,370).

Overall, 3 (3.8%) dogs experienced local recurrence after 17, 64, and 150 days. In one of them, the cancer was invading the bone. None of these dogs received further treatment other than excision of the primary SCC.

Eighteen (22.8%) dogs developed *de novo* SCCs after a median of 190 days (range, 30–1,688). Among them, 6 received adjuvant medical treatment, consisting of 5-Fluorouracil (*n* = 3), carboplatin (*n* = 1), cisplatin (*n* = 1), and metronomic therapy (*n* = 1).

Fourteen (17.7%) dogs developed metastatic disease during the follow-up after a median of 266 days (range, 36–1,070). Metastatic sites included lung (*n* = 7), regional lymph nodes (*n* = 5), skin (*n* = 1), and kidneys and liver (*n* = 1). All metastasis were confirmed by means of cytological evaluation, excluding the pulmonary, which were supposed based on thoracic radiographs. Among dogs developing metastases, 7 (50%) already had nodal metastatic disease at presentation and failed at another site. Only 4 of them had received adjuvant chemotherapy, consisting of carboplatin (*n* = 4) and cisplatin (*n* = 1).

At the end of the study, 24 dogs had died because of progressive disease after a median of 302 days (range, 10–1,468).

Thirty-one dogs had died due to tumor-unrelated causes after a median of 745 days (range, 128–2,370) and 24 dogs were still alive after a median follow-up of 432 days (range,147–2,254).

Overall, the median TTP and ST time of the dogs in the study were not reached. The 1- and 2-year survival rates were of 81 and 60%, respectively.

Results of univariable survival analysis are reported in Table 2.

**Table 2:** Univariable Cox regression analysis of variables potentially associated with increased risk of tumor progression, *de novo* tumor development and survival time in 79 dogs with digital squamous cell carcinoma.

**Variable**	**Tumor progression**		**Development of new digital lesions**		**Tumor-related death**	
	**HR (95% CI)**	***P***	**HR (95% CI)**	***P***	**HR (95% CI)**	***P***
Breed	1.8 (0.9–3.7)	0.113	2 (0.8–5)	0.159	3.1 (1.4–7)	0.006^*^
Schnauzer						
Other^**^						
Sex	1.9 (0.8–4.2)	0.134	1.8 (0.6–5)	0.282	1.5 (0.6–3.6)	0.363
Male						
Female^**^						
Age^a^	1.4 (0.6–2.8)	0.410	1.3 (0.5–3.4)	0.561	2.1 (0.8–4.9)	0.094
≤ 10 years^**^						
>10 years						
Weight^a^	1 (0.5–2.1)	0.967	0.7 (0.3–1.7)	0.407	0.9 (0.4–2.1)	0.877
≤ 32 kg^**^						
>32 kg						
Duration of symptoms^a^	2.4 (1.1–5.1)	0.02^*^	2.4 (0.9–6.3)	0.081	1.8 (0.8–4.2)	0.141
≤ 90 days^**^						
>90 days						
Presentation	2.9 (1–8.5)	0.047^*^	5.8 (1.9–18.1)	0.002^*^	2 (0.7–5.9)	0.199
Single^**^						
Multiple						
Subungual	3 (0.9–10)	0.072	5.4 (0.7–40.7)	0.104	1.4 (0.5–4.3)	0.511
Yes						
No^**^						
Ulceration	1.3 (0.6–2.9)	0.467	1.1 (0.4–2.9)	0.811	1.8 (0.7–4.4)	0.216
Yes						
No^**^						
Tumor diameter^a^	1.5 (0.6–4.2)	0.391	1.5 (0.4–5.5)	0.559	1.1 (0.4–3.1)	0.839
≤ 2.3 cm^**^						
>2.3 cm						
Bone lysis	0.9 (0.2–4)	0.930	1.2 (0.2–9.5)	0.830	0.7 (0.1–3.0)	0.628
Yes						
No^**^						
Metastasis at admission	2.2 (0.5–9.1)	0.294	0.5 (0 ≥ 100)	0.627	7.1 (2.4–21.4)	<0.001^*^
Yes						
No^**^						
LNctomy	1.2 (0.6–2.6)	0.568	1.2 (0.5–3.1)	0.660	1.6 (0.7–3.6)	0.287
Yes						
No^**^						
Adjuvant medical treatment	4 (1.8–8.4)	0.001^*^	4.3 (1.6–11.3)	0.003^*^	2.9 (1.3–6.7)	0.012^*^
Yes						
No^**^						

*LNctomy, lymphadenectomy; HR, hazard rato; CI, confidence interval.*

a *Median used as cut-off value.*

* *Significant.*

** *Reference category*.

Variables significantly associated with tumor progression included the duration of symptoms >90 days (HR = 2.4; 95% CI = 1.1–5.1; *P* = 0.02), involvement of multiple digits (HR = 2.9; 95% CI = 1–8.5; *P* = 0.047) and the administration of adjuvant medical treatment (HR = 4; 95% CI = 1.8–8.4; *P* = 0.001).

Variables significantly associated with the development of new digital lesions included involvement of multiple digits at presentation (HR = 5.8; 95% CI = 1.9–18.1; *P* = 0.002) and the administration of adjuvant medical treatment (HR = 4.3; 95% CI = 1.6–11.3; *P* = 0.003).

Variables significantly associated with tumor-related death included presence of metastatic disease at admission (HR = 7.1; 95% CI = 2.4–21.4; *P* < 0.001), the Schnauzer breed (HR = 3.1; 95% CI = 1.4–7; *P* = 0.006), and the administration of adjuvant medical treatment (HR = 2.9; 95% CI = 1.3–6.7; *P* = 0.012).

On multivariable analysis, only adjuvant medical treatment retained significance for tumor progression; whereas all the aforementioned variables confirmed their significance for new lesion development and tumor-related death (Table 3).

**Table 3:** Multivariable Cox regression analysis of variables potentially associated with increased risk of tumor progression, *de novo* tumor development and survival time in 79 dogs with digital squamous cell carcinoma.

	**Covariates**	**HR (95% CI)**	***P***
Tumor progression	Duration of symptoms >90 days^a^	1.7 (0.7–3.8)	0.216
	Multiple tumors at presentation	1.5 (0.4–5.4)	0.520
	Adjuvant medical treatment	2.9 (1.2–7.2)	0.018^*^
Development of new digital lesions	Multiple tumors at presentation	4 (1.2–13.2)	0.023^*^
	Adjuvant medical treatment	3.4 (1.2–9.4)	0.019^*^
Tumor-related death	Schnauzer breed	3.9 (1.7–9.2)	0.002^*^
	Metastasis at admission	5.8 (1.8–18)	0.002^*^
	Adjuvant medical treatment	2.8 (1.2–6.9)	0.021^*^

*HR, hazard rato; CI, confidence interval.*

a *Median used as cut-off value.*

* *Significant*.

## Discussion

Squamous cell carcinoma develops following malignant transformation of specialized keratinocytes and is the most common cancer of the digit in dogs (1, 2). Despite this, no updated literature is available. Here, we report our experience regarding presentation, biological behavior and prognosis of dogs with digital SCC, highlighting the therapeutic approach and giving a better insight into prognostic factors.

In the current retrospective multicenter study, 79 dogs with digital SCC were included. Based on our findings, we confirm that dark-coated dogs were over-represented similarly to earlier findings, with Schnauzers representing approximately one third of the population, further implicating a possible familial association and/ or genetic pre-disposition as previously described (12, 16, 17). Additionally, according to the univariate analysis, the Schnauzer breed was linked to a higher risk of tumor-specific death compared with all other breeds. The reason behind this remains unknown. Schnauzers did not have any apparent poor prognostic factors at presentation, therefore the clinical behavior may be influenced by breed, as documented for other tumors such as mast cell tumors (18). Conversely, the association may just be a coincidental finding.

Similarly to previous reports (1), the majority of SCCs occurred in the front limbs (61% of cases), although they can develop in any digit.

It has been previously documented that subungual SCCs are associated with a better outcome (14). In the current case series, 75% of dogs had their SCC arising from the subungual epithelium, yet this was not linked to a longer survival. The small number of dogs having their SCC arising at sites different from the nail bed may have prevented a possible prognostic association to emerge.

At admission, ~9% of dogs had multiple digit involvement, and this was associated with a shorter TTP and with the development of new digital lesions. Synchronous SCCs are an exceptional presentation in people (<4% of cases) (19), with trauma, PV infection and occupational exposure possibly acting as predisposing risk factors in some cases (4).

In past studies, presence or development of new digital lesions was mentioned in 3/33 dogs with SCC according to one study (1); 13/154 had multiple digit involvement at presentation and 11/49 developed *de novo* digital lesions in other digits according to another (10). However, no risk factors were identified (1, 10). The finding that multiple digit involvement poses a higher risk is therefore new and important. Owners should be informed that—if synchronous lesions are present—their dogs may develop further lesions in the future.

Additional dogs and tumor variables such as sex, age, weight, ulceration, tumor diameter, and bone lysis did not show statistically significant impact on TTP, development of new digital lesions and ST.

Digital SCC can be difficult to diagnose because its presentation can mimic other conditions (9). A duration of symptoms >90 days was significantly associated with higher risk for tumor progression. The diagnostic delay is common because clinical signs are initially mild and may be disregarded at first. Also, as anticipated, clinical similarities to many inflammatory and benign neoplastic conditions may cause diagnostic confusion and further delay in diagnosing the disease. Indeed, digital SCC often presents with non-specific clinical features such as pain, disruption of the nail, ulceration, periungual or subungual mass, paronychia, destruction of the nail or onychomadesis. As a consequence, the delay in diagnosis could lead to more advanced disease states or higher metastatic rate at presentation and could be a reason why the longer duration of clinical signs was associated with a worse outcome.

Although chronic inflammation and infections have been suggested as etiological factors triggering neoplastic transformation in human SCC (20), thereby triggering neoplastic transformation, it remains unclear whether the same holds true for dogs with digital SCC.

Regardless, a biopsy in every digit abnormality not responding to initial treatment should be carried out to avoid delays in diagnosis and subsequent treatment. Radiographs should also be obtained, as bone involvement occurred in >90% of dogs in the current series population and helps guide the extent of surgery.

The overall metastatic rate at admission was 15.2%, in line with the published literature, and none of the dogs had distant metastasis (1, 14). Thoracic radiographs and abdominal ultrasound were carried out in the majority of dogs, highlighting that there is little evidence on the need for advanced imaging investigations to detect metastases in digital SCC. Based on the previous and current findings, digital SCC can most likely be considered as a malignancy with a low tendency to metastasize if treated adequately. Nevertheless, assessment of the regional lymph nodes as routine examination should be performed.

All dogs in the current study underwent cytological evaluation of the regional lymph node, and 4 (5.1%) had evidence of metastatic disease. Approximately 50% of dogs also underwent lymphadenectomy, and metastatic disease was detected in 6 additional dogs, leading to an overall nodal metastatic rate at presentation of 12.7%. This finding emphasizes the need for nodal histopathological evaluation to detect metastasis, as nodal metastases worsened outcome in the current case series. Also, cytology might not be able to rule out early metastatic disease in all cases.

Lymphadenectomy *per se* did not show any statistically significant impact on TTP, development of new digital lesions and ST, thereby not having a therapeutic implication. However, it must be acknowledged that 50% of dogs did not have the regional lymph node removed, possibly contributing to an outcome bias. Future prospective studies must address whether lymphadenectomy of metastatic nodes improves survival.

Given the rarity of digital SCC, there is no consensus on the optimal treatment. No standardized therapeutic approach is described in SCC, and the choice is selected on the basis of the extent of the tumor.

Complete surgical excision was obtained in all dogs; there were 3 (3.8%) cases with local recurrence after amputation. Thus, it appears that digit amputation may achieve adequate local tumor control in the majority of cases.

Adjuvant treatments, including chemotherapy and radiation therapy, were only carried out in 21.5% of dogs. According to our data analysis, adjuvant chemotherapy was associated with a worse outcome. It is plausible that this is a selection bias, with dogs with more extensive disease and thus a worse prognosis more likely treated with chemotherapy. Due to the relatively non-aggressive clinical behavior and low metastatic rate, chemotherapy should most likely only be offered in the case of metastatic disease. Nevertheless, the best protocol has not been established yet.

Eighteen (22.8%) dogs developed *de novo* SCCs during the follow-up period. Thus, owners need to be instructed to check their dog's nails routinely and to seek for professional guidance as soon as any clinical changes are noted. Moreover, careful examination of the digits upon annual veterinary check-ups should be encouraged for breeds at high risk for digital SCC.

Overall, 17.7% of dogs developed metastatic disease during the follow-up; half of them had nodal metastasis at admission. It may be concluded that dogs with lymph node metastasis are at risk for further metastases and should be monitored more closely.

In line with the published literature, the 1- and 2-year survival rates in the current study were of 81 and 60%, respectively, whereas the median TTP and ST time were not reached.

This study has several limits, mainly attributable to its retrospective nature.

Different imaging modalities were carried out during the initial staging work-up, and it may be possible that pulmonary metastases were missed in those cases undergoing thoracic radiographs. Nevertheless, the long follow-up would have made it possible to identify dogs with lung metastases.

Lymphadenectomy was only performed in 50% of dogs, thereby precluding to draw any meaningful conclusions regarding its therapeutic role. Also, adjuvant treatment was not standardized, and the motivation behind the choice as to whether or not administering chemotherapy was unclear.

None of the cases had review of the histopathological findings, therefore precluding us from identifying other variables that may help in predicting cases prone to more aggressive biological behavior. Immunohistochemical assessment of adhesin molecule such as E-cadherin, β-catenin and desmoglein in combination with invasive front grading of the tumor, may be used to predict the biological behavior of canine oral and cutaneous SCC (21). In the aforementioned study, higher histological grade was associated with a more aggressive biologic behavior. In the current study, immunohistochemistry was not performed in any case.

Nevertheless, only 4 SCCs were classified as poorly differentiated. Considering the above, the utility of immunohistochemistry in predicting the biological behavior of digital SCC in the present study is questionable.

This study, however, is unique in that only those cases that had a complete work-up, an accurate treatment and outcome description and an adequate follow-up were included in the analysis.

In conclusion, the diagnosis of digital SCC can be challenging, as it is often delayed by the presence of clinical signs non-specific of neoplasia, encompassing a variety of differential diagnosis of nail and limb pathologies. Although, metastases remain rare with prolonged TTP and disease-specific ST, non-resolving digit and nail conditions, especially if present in dark-coated dogs, should raise the suspicion of digital SCC and investigations should be directed accordingly. Multiple lesions and nodal involvement at diagnosis are associated with a poorer outcome. Schnauzers remain a predisposed breed and with the potential of a poorer clinical outcome, whereas non-subungual origin did not seem to impact outcome in this cohort of patients. Digital amputation is still the treatment of choice and the majority of dogs will benefit from a surgical procedure as single treatment modality. Regional lymphadenectomy can offer important prognostic information and we advise to include this in the surgical treatment plan.

## Data Availability Statement

The raw data supporting the conclusions of this article will be made available by the authors, without undue reservation.

## Ethics Statement

Ethical review and approval was not required for the animal study because retrospective study. Written informed consent was obtained from the owners for the participation of their animals in this study.

## Author Contributions

LM conceived the presented idea and planned the study. LM, DM, EM, LP, AF, DG, MAr, VM, RF, CC, PM, MAn, and FA collected the data and SS performed the statistical analysis and the data interpretation. LM and SS drafted the manuscript and prepared the tables and the figures. LM, RF, DM, VM, and SS revised the manuscript. All authors discussed the results and approved the final version of the manuscript.

## Conflict of Interest

The authors declare that the research was conducted in the absence of any commercial or financial relationships that could be construed as a potential conflict of interest.
